# Physical Activity, Energy Expenditure, Screen Time and Social Support in Spanish Adolescents—Towards an Explanatory Model about Health Risk Factors

**DOI:** 10.3390/ijerph191610222

**Published:** 2022-08-17

**Authors:** Daniel Sanz-Martín, Eduardo Melguizo-Ibáñez, Germán Ruiz-Tendero, Félix Zurita-Ortega, José Luis Ubago-Jiménez

**Affiliations:** 1 Faculty of Humanities and Social Sciences, Isabel I University, 09003 Burgos, Spain; 2 Department of Didactics Musical, Plastic and Corporal Expression, Faculty of Education Science, University of Granada, 18071 Granada, Spain; 3 Department of Languages, Arts and Physical Education Teaching, Faculty of Education, Complutense University of Madrid, 28040 Madrid, Spain

**Keywords:** light physical activity, moderate–vigorous physical activity, social support, screen time, adolescent

## Abstract

Youth obesity has been a pandemic for decades. One of its causes is a low level of physical activity. It is necessary to know the specific situation of adolescents and the factors that influence it in order to be able to act accordingly. The first aim of the current study is to create an explanatory model to establish the relationships between light physical activity time, light physical activity energy expenditure, screen time and social support. The second aim is to propose a theoretical model specifying the relationships between moderate–vigorous physical activity time, moderate–vigorous physical activity energy expenditure, screen time and social support. The study design was non-experimental (ex post facto), descriptive-correlational and cross-sectional. A total of 694 adolescents from the region of Soria (12–17 years) participated in the study. The instruments administered were the Four by One-Day Physical Activity Questionnaire, Parent Support Scale and Peer Support Scale. Two structural equation models were developed to analyse the relationships between the variables that comprised the explanatory models. The results show that social support had a negative influence on screen time in the proposed model in relation to light physical activity (r = −0.210; *p* ≤ 0.001) and in the proposed one regarding moderate–vigorous physical activity (r = −0.173; *p* ≤ 0.05). Social support was negatively related to light physical activity time (r = −0.167; *p* ≤ 0.05). Family support had a greater influence than did peer support. In conclusion, the models for light and moderate–vigorous physical activity are useful to describe the relationships between time, energy expenditure, screen time and social support.

## 1. Introduction

Global obesity has tripled since 1975, with more than 340 million children and adolescents overweight or obese in 2016 [1]. Moreover, levels continue to rise [2]. Sedentary lifestyles and eating habits are two of the causes of this pandemic situation [2,3]. Because of this, health and physical activity (PA) are one of the issues of greatest concern in today’s society [4,5]. This concern is to some extent due to the fact that PA practice has numerous health benefits [6,7], which include the prevention of overweight/obesity [8]; however, the current levels of practice are low [1,4,5]. For PA to be beneficial, it should be performed according to recommendations, which for children and adolescents is at least 60 min/day of moderate–vigorous physical activity (MVPA) [5,9,10,11].

Despite social concern and evidence regarding the benefits of PA practice, more than 70% of adolescents are inactive [12,13], i.e., they do not meet PA practice recommendations [9]. Moreover, these levels decrease with age, with a critical period of decline around the age of 9 years, which is more pronounced in girls than in boys [14]. These levels have worsened since the beginning of the pandemic caused by COVID-19 [15,16].

In order to be able to go deeper into the problem of low levels of physical activity in adolescents, it is necessary to know the types of PA according to their intensity and the factors that condition it. On the one hand, PA can be classified according to its intensity by expressing it in metabolic equivalents (METS). For this purpose, a METS is considered to be the energy equivalent expended by an individual while seated at rest [9]. MVPA is that type of activity that involves an expenditure of at least 3 METS/h.

If it is less than 3 METS, it is considered as light physical activity (LPA) [17]. On the other hand, PA practice is conditioned by several correlates, which vary in type and intensity, depending on age. In adolescents, sedentary behaviour outside the school day and social support (SS) from parents and significant others are among the most significantly influential determinants, with negative and positive relationships, respectively [18,19].

Adolescents spend part of their leisure time in sedentary activities [20], such as screen-based activities (e.g., playing computer or video games or watching TV) [21]. The time spent on these activities exceeds the WHO maximum recommended time of 2 h per day [22,23,24]. In some cases, it even exceeds the time spent on PA [25]. Furthermore, the relationship between PA and screen time (ST) is negative and significant [18,26,27].

SS perceived by adolescents from parents and peers have the greatest influence on their PA levels [28]. Moreover, the influence they perceive from peers is mostly higher than the influence from their parents, although this varies by country [29]. In any case, the relationship between PA and SS is positive and significant [18,19,30].

Although there is evidence on the relationship between PA and ST, as well as PA and SS, no studies have been found in which a model has been presented that justifies the relationships between the three elements for the adolescent population through direct measurements. In this model, SS could play an important role [31]. The existence of such a model would help to better understand the reality of a population at a given time. In this way, it would be possible to better adapt the actions needed to improve PA levels [32]. Moreover, it would be interesting to know how this model performs depending on the type of PA intensity.

Based on the above, our study was proposed with the following objectives: (1) to create an explanatory model to establish the relationships between LPA time, LPA energy expenditure, screen time and social support of adolescents and (2) to propose a theoretical model that specifies the relationships between MVPA time, MVPA energy expenditure, screen time and social support of young people.

## 2. Materials and Methods

### 2.1. Design and Subjects

The study is framed within the physical activity and health paradigm [33] and behavioural epidemiology [34]. Furthermore, the method is non-experimental (ex post facto), cross-sectional, descriptive and correlational [35] of physical activity, social support and screen time in Spanish adolescents.

The research involved adolescents from Soria (Spain) aged between 12 and 17 years (14.06 ± 1.27). The population in the region of Soria is 3224 people. The sample study was non-probabilistic and by convenience. The final sample was 694 people, which means a precision error of 3.3%. According to sex, 364 were boys (52.4%) and 330 were girls (47.6%). Out of 19 schools, 17 schools agreed to participate, from each of which a class group of students was selected as potential participants. The criterion of accessibility was followed for the selection of the groups, favouring that those from each centre could answer the questionnaires on the same days.

### 2.2. Instruments and Variables

The use of the Four by One-Day Physical Activity Questionnaire (FBODPAQ) has made it possible to measure physical activity levels. This instrument was designed by Cale [36] for British adolescents, adapted to Spanish by Cantera [37], and it was validated for the same population by Soler et al. [38]. The reliability obtained in Cronbach’s alpha was α = 0.832. Subsequently, it has been used in other research [4,39,40,41].

The variables obtained from FBODPAQ were PA and ST. PA was differentiated according to the intensity in LPA (<3 METS/h) and MVPA (≥3 METS/h), expressed in minutes and energy expenditure (EE). In addition, ST was calculated by adding the time reported by adolescents in the items “watching TV” and use of “computer, video games and internet”.

The Parent Support Scale and Peer Support Scale were used to measure social support for PA practice. The Spanish version [4] of the one originally designed by Prochaska et al. [42] was used. The scales ask about the support received during the last seven days. Both instruments consist of five Likert-scale items ranging from 0–4, where 0 means not at all, and 4 means every day. The reliability of the items ranged between 0.7 and 0.83 Cronbach’s alpha. These scales have already been used in other studies [43,44].

### 2.3. Procedure

The study began by performing a documentary search on the research topic. Afterwards, the research project was drafted, focused on investigating the relationships between PA, ST and SS of adolescent pupils. This was a novelty, as no previous study was found for this purpose.

The research project was based on the ethical principles established in the Declaration of Helsinki. Furthermore, it was approved by the Ethics Committee of the University of Granada (1478/CEIH/2020). In addition, permission for access to the educational centres was obtained from the regional director of education in Soria. In addition, an informed consent form was provided in advance to the adolescents. This had to be signed by their legal guardians and delivered to the research team before the first day of administration of the instruments.

Subsequently, the data obtained were analysed statistically and linked to the previously existing scientific evidence.

### 2.4. Data Analysis

The statistical software IBM SPSS Statics 26.0 (IBM Corp, Armonk, NY, USA) was used to create the data matrix and perform the descriptive analysis. The Kolmogórov-Smirnov test was used to check that the variables followed a normal distribution. In addition, Cronbach’s Alpha test was applied to calculate the reliability of the research instruments.

**Figure 1:**
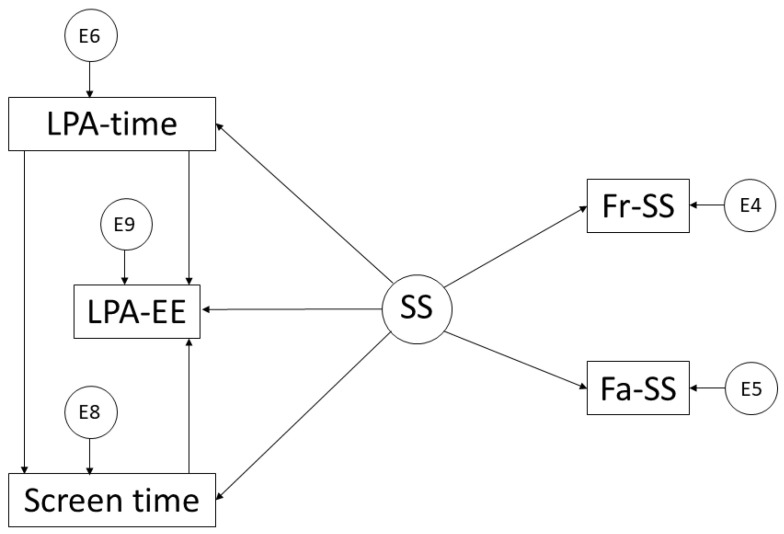
Theoretical model proposed in relation to light physical activity. Light Physical Activity Time (LPA-time); Light Physical Activity Energy Expenditure (LPA-EE); Screen Time (ST); Social Support (SS); Support from Friends (Fr-SS); and Support from Family (Fa-SS).

**Figure 2:**
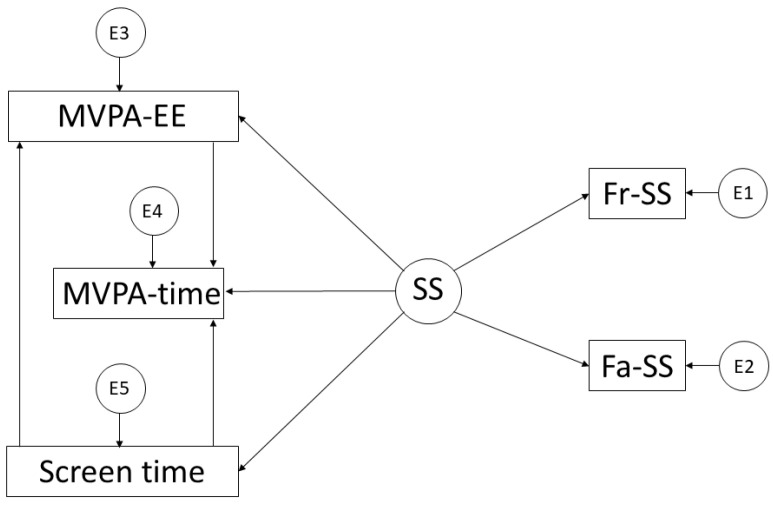
Theoretical model proposed in relation to MVPA. Moderate–Vigorous Physical Activity Time (MVPA-time); Moderate–Vigorous Physical Activity Energy Expenditure (MVPA-EE); Screen Time (ST); Social Support (SS); Support from Friends (Fr-SS); and Support from Family (Fa-SS).

In addition to the previous software, the IBM SPSS Amos 26.0 (IBM Corp, Armonk, NY, USA) was used. This allowed us to create the structural equation models, and as a consequence, to be able to analyse the relationships between the variables that made up the theoretical model. One of these models (Figure 1) includes five observed or endogenous variables: EE in LPA, time in LPA, ST, parental support and support from friends. In addition, the unobserved or exogenous variable SS was included. In the other model (Figure 2), the same variables were used; however, those relating to EE and time were derived from MVPA. 

We decided to analyse the relationship of variables in two theoretical models, one for LPA and the other for MVPA because the factors that influence each type of PA and the health outcomes are different. This is because the scientific evidence mentions that MVPA has greater health benefits—for example, reducingthe risk factors associated with cardiovascular disease or obesity [17]. In addition, international practice recommendations are relative to the time of performing MVPA [5,9,10,11]. 

In contrast, LPA has fewer health effects, is not counted in compliance with PA practice recommendations, and is linked to sedentary activities [17]. With respect to the endogenous variables that comprise the models, the measurement error of these variables is incorporated. This is a consequence of the causal explanation of the observed associations between indicators and measurement reliability. In addition, the one-way arrows represent the lines of influence between the latent variables. This allows for their interpretation with the incorporation of the regression weights.

Finally, the fit of the proposed models was assessed. The goodness of fit has to be evaluated with respect to Chi-Square, where a correct fit was obtained based on the associated *p*-values to be non-significant [45,46]. Likewise, the comparative fit index (CFI) has to be higher than 0.95, with a normal fit index (NFI) score higher than 0.90, incremental fit index (IFI) higher than 0.90, Tucker–Lewis index (TLI) higher than 0.90 and root mean square error of approximation (RMSEA) lower than 0.1 [47,48].

## 3. Results

The model developed through the variables in a representative sample of adolescents in the region of Soria shows a good fit for each of the indices that comprise it. Focusing attention on the model developed for the practice of light physical activity showed a significant *p*-value (X^2^= 8.489; df = 2; pl = 0.014). However, due to the influence of sample susceptibility and sample size, the data cannot be interpreted in an independent way [49]; therefore, other standardised fit indices have been used. In this case, the CFI scored 0.993, the NFI reflected a value of 0.991, the IFI showed a score of 0.993, the TLI showed a score of 0.965, and finally the RMSEA reflected a score of 0.095.

**Figure 3:**
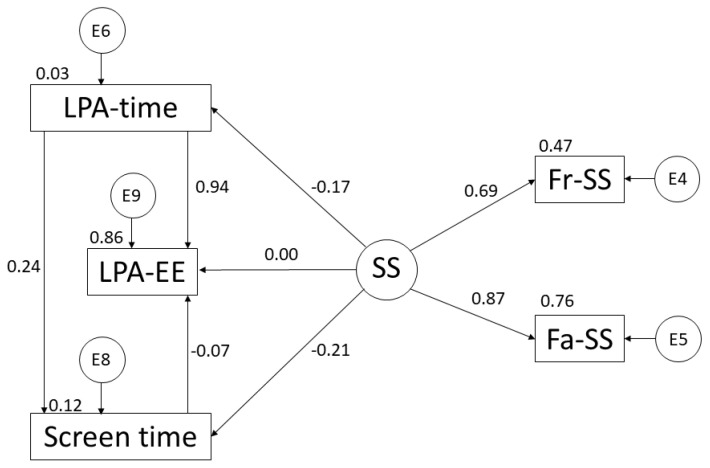
The proposed model in relation to light physical activity. Light Physical Activity Time (LPA-time); Light Physical Activity Energy Expenditure (LPA-EE); Screen Time (ST); Social Support (SS); Support from Friends (Fr-SS); and Support from Family (Fa-SS).

In this case, focusing on what is shown in Table 1 and Figure 3, the SS variable shows negative relationships with the practice of LPA (r = −0.167; *p* < 0.05) and with ST (r = −0.210; *p* < 0.001). However, positive relationships are observed with support from friends (r = 0.686), with family support (r = 0.871; *p* < 0.001) and with EE (r = 0.001). Following with the time spent practising LPA, a positive relationship was observed with ST (r = 0.239; *p* <0.001) and with EE (r = 0.944; *p* < 0.001). Finally, regarding the relationship between EE and ST, a negative relationship was observed (r = −0.067; *p* < 0.001).

Proceeding with the model developed for MVPA, a good fit is observed for each of its component indices. In this case, the Chi-Square showed a significant *p*-value (X^2^ = 1.236; df = 2; pl = 0.539). Likewise, the CFI scored 0.999, the NFI reflected a value of 0.999, the IFI score was 0.991, the TLI showed a score of 0.994, and finally the RMSEA reflected a score of 0.004.

**Figure 4:**
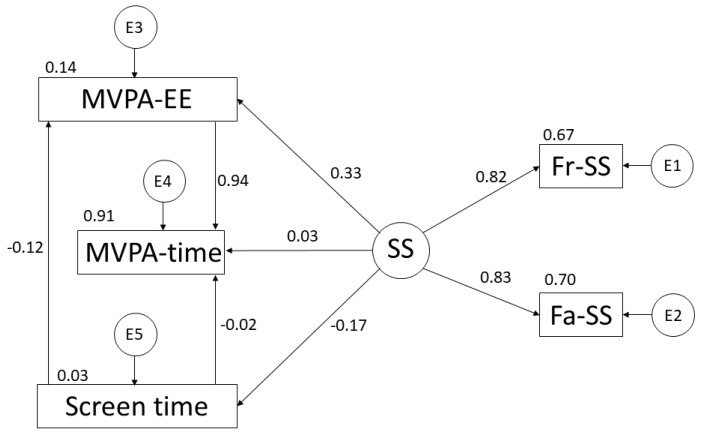
The proposed model in relation to moderate–vigorous physical activity. Moderate–Vigorous Physical Activity Time (MVPA-time); Moderate–Vigorous Physical Activity Energy Expenditure (MVPA-EE); Screen Time (ST); Social Support (SS); Support from Friends (Fr-SS); and Support from Family (Fa-SS).

In this case, Figure 4 and Table 2 show the existing relationships for participants practising MVPA. Focusing attention on SS, a negative relationship with ST is observed (r = −0.173; *p* < 0.05). However, positive relationships are shown with EE (r = 0.328: *p* < 0.001), support from friends (r = 0.818), support from family (r = 0.834; *p* < 0.001) and time spent practising MVPA (r = 0.033). Continuing with ST, negative relationships are shown with EE (r = −0.120; *p* < 0.05) and time of MVPA (r = −0.015). Finally, a negative relationship is shown between ST and EE (r = −0.120; *p* < 0.05).

## 4. Discussion

The theoretical models presented serve to explain the relationships between SS, the dimensions (time and EE) of PA and ST of adolescents in the region of Soria. There are significant differences between the LPA model and the MVPA model. Next, we will compare both models and proceed to a discussion based on the scientific literature.

In both models presented in this study, the importance of social support as a determinant of physical activity is perceived. With respect to the LPA model, SS was slightly, negatively and significantly related to LPA time. In contrast, the relationship between SS and EE in LPA was positive and not significant. Other links are also observed in the MVPA model. The relationship between SS and MVPA time was positive and not significant. In contrast, the relationship between SS and EE in MVPA was positive but significant.

The differences in the relationships found between SS and PA may be due to the way in which the EE of PA is calculated as a function of intensity. In FBODPAQ, five categories of PA are differentiated [36,37,38]: very mild (1.5 METS/h), mild (2.5 METS/h), moderate (4 METS/h), severe (6 METS/h) and very severe (10 METS/h). Therefore, the intensity stipulated in the questionnaire protocol was considered for the calculation of EE. 

In contrast to this, for the present study, and based on the trend in the scientific literature [5,9,10,11,17], we decided to regroup the categories nominally into two: LPA (<3 METS/h) and MVPA (≥3 METS/h) as in previous studies [50,51]. However, this was not the case for the time computation, as it was the sum of PA times according to intensities. 

As a consequence, it can be deduced that the activities with the highest average METS of each of the source categories substantially influenced the EE calculation, with those of high intensity playing a particularly important role. Likewise, it would be convenient to differentiate the type of PA intensity when measuring the degree of compliance with the practice recommendations. Furthermore, this explanation justifies the relationships between time and EE for both LPA and MVPA, with both being almost perfect and highly significant.

Little evidence has been found to compare the relationship of SS and time in LPA obtained with that of other studies. Lawman and Wilson [52] found that parental SS was positively and significantly related to the LPA of obese underserved adolescents. This relationship with MVPA was positive but not significant. In addition, Huffman et al. found that tangible parental support was positively related to minutes of LPA [53]. The relationship between SS and MVPA time of the Soria adolescents is similar to that found in other studies, being positive. 

The study by Wang et al. [54] showed that higher levels of family support in Chinese adolescents were less likely to have insufficient PA. Engels et al. [55] found a positive relationship between friends’ support and MVPA in Hawaiian adolescents. Furthermore, in the case of family support, the relationship was significant. Pluta and colleagues [56] also found positive and significant relationships in both cases and even with teacher support in adolescents from Wielkopolska (Poland). Although it was not considered in the study, it should also be considered that not all types of social support are equally influential. Tangible support appears to be one of the most important [57].

In this study, conducted with an adolescent population in Soria, it can be observed that the relationship between SS for PA and ST was negative, weak and significant. This is true for both explanatory models. This result was similar to the study by Park and Park [58] regarding the relationships of parental SS for PA, PA, ST and body weight of US high-school students in an explanatory model. 

Costigan et al. [59], in a review of an adolescent female population, highlighted three of the four studies on the subject. They found a negative relationship between screen-based sedentary behaviour and socialising/SS, one of which was significant. These authors highlighted that three of the four studies on the subject found a negative relationship between screen-based sedentary behaviour and socialising/SS, one of which was significant. In contrast, the other study had a positive and significant relationship.

Although the relation between SS and PA was evident, as well as SS and ST, it can be seen how, in the two models obtained, family support had a greater relation with the latent variable SS than friends, which was positive and significant. The type of relationship is in line with the results of previous studies but with neither the degree nor the predominance. Haidar et al. [60] found that the SS of friends was a better predictor of moderate PA, vigorous PA and PA than the SS of parents. 

Regardless of the type of support, the predictions were significant. The same was true for ST prediction but not significantly so. Lisbon and colleagues [15] obtained similar results to those of the study by Haidar et al. In this study, the structural equation model showed a greater relationship of PA with SS from friends than from family. In relation to the rural Chinese youth population [61], support from families was higher than from friends. However, with respect to relationships with PA, support from friends was more related to exercise intensity and time but not to frequency. Regardless of the variables, all relationships were positive and significant.

Perhaps the difference in the predominance of the agent that most influences SS is due to the fact that in the other studies the relationships between PA and SS were analysed without considering the influence of the variable ST in this relationship. It could also be that there were other variables not considered in any of the studies that influenced these relationships. This difference could even be due to the fact that, in the present study, SS was calculated as the mean score of the items instead of considering them independently.

In this study with a population from Soria, different relationships were obtained between ST and PA. Time in LPA was positively, slightly and significantly related to ST. In contrast, the relationship with time in MVPA was negative, slight and non-significant. On the other hand, a similar relationship was observed as a function of EE. Although the relationship between MVPA-EE and ST was slightly higher, both MVPA-EE and ST were negative, slight and significant. These differences in the relationships between time and EE may be due to the fact that the original subcategories of the questionnaire “very light PA” and “very vigorous PA” are the most negatively related to ST and, therefore, the ones that most condition the newly created categories LPA and MVPA.

These results are similar to those found in previous studies. O’Brein et al. [24] also found negative, but non-significant, relationships between MVPA and overall TS in a sample of Irish adolescents as a function of gender. Furthermore, these relationships were similar in moderate PA and vigorous PA. Braig et al. [27] found that the leisure time PA of 13-year-old adolescents was negatively related to different types of screen activities, with the exception of TV viewing, which was positively related. 

These relationships were non-significant, irrespective of the sex of the young people. Costigan et al. [59] also found that the majority of studies (60%) selected in their systematic review demonstrated a negative association between PA/fitness and screen-based sedentary behaviour. McVeigh and Meiring [62] found that, in a sample of South African adolescents, PA decreased with age while screen time increased, although no relationship was found between the two variables.

In this study, SS is a determinant of models, including LPA, MVPA and ST variables. Furthermore, SS was shown to be a mediator between physical exercise and social anxiety [61], and thus it could be a mediator of other factors as well.

In the following, the limitations of the study will be discussed. Although the results are generalisable to the adolescent population of the region of Soria, they cannot be extrapolated to the adolescent population worldwide. As shown, the determinants that condition PA vary according to the characteristics of the population, both in type and intensity. Another limitation is due to the use of the FBODPAQ. This instrument asks about the previous day’s physical activity and was administered over four days. 

In addition, several of the studies cited in this paper used questionnaires that asked for PA over the last seven days. This makes it easier to compare the PA levels more objectively. Thirdly, ST was calculated as the sum of FBODPAQ item scores (TV viewing and use of “computer, video games and internet”). This implies that activities, such as mobile phone and tablet use, were not considered. 

There is the limitation that there could be a variable not considered in the study that has a direct influence on those considered and that could modify the contrasted models.

Finally, future lines of research will be proposed. It would be interesting to conduct similar studies with adolescents from other cities and countries, in order to be able to compare the results. It would also be possible to extend the age of the participants and analyse the evolution in a longitudinal study. Knowing how each type of SS of the different agents influences similar models would be useful for adopting more effective measures to promote PA.

## 5. Conclusions

The first theoretical model presented is useful for explaining the relationships between the time of LPA, EE of LPA, ST and SS of adolescents in the region of Soria. This was also corroborated for the model relating to MVPA. This is because, in both cases, the general equations include parameters with acceptable values.

SS was a determining factor in both explanatory models. It was negatively related to LPA time and ST in the first model and ST in the model relative to MVPA. In addition, SS was related to time in MVPA, but positively and, unlike the rest of the previous links, it was not significantly related to time in MVPA.

LPA practice time was positively related to ST. On the other hand, time spent in MVPA was negatively related, although in this case, it was not significantly so. The family was the agent that most influenced the mean levels of SS, regardless of the PA intensity model. The difference between relationships was greater in the LPA model.

## Figures and Tables

**Table 1:** The model developed for light physical activity.

Associations between Variables	R.W.				S.R.W.
	Estimations	S.E.	C.R.	*p*	Estimations
LPA Time ← SS	−29.216	10.383	−2.814	**	−0.167
ST ← SS	−41.615	11.592	−3.590	***	−0.210
ST ← LPA time	0.272	0.058	4.722	***	0.239
Fr-SS ← SS	1.000				0.686
Fa-SS ← SS	1.732	0.405	4.282	***	0.871
LPA-EE ← SS	0.005	0.125	0.040	0.968	0.001
LPA-EE ← LPA time	0.029	0.001	45.854	***	0.944
LPA-EE ← ST	−0.002	0.001	−3.172	**	−0.067

Regression Weights (R.W.); Standardised Regression Weights (S.R.W.); Standard Error (S.E.); Critical Ratio (C.R.); Light Physical Activity Time (LPA Time); Social Support (SS); Screen Time (ST); Support from Friends (Fr-SS); Support from Family (Fa-SS); and Energy Expenditure on Light Physical Activity (LPA-EE); *** *p* ≤ 0.001; ** *p* ≤ 0.05.

**Table 2:** The model developed for moderate–vigorous physical activity.

Associations between Variables	R.W.				S.R.W.
	Estimations	S.E.	C.R.	*p*	Estimations
MVPA-time ← SS	2.611	1.633	1.598	0.110	0.033
ST ← SS	−22.871	8.179	−2.796	**	−0.173
MVPA-time ← ST	−0.009	0.010	−0.893	0.372	−0.015
Fr-SS ← SS	1.000				0.818
Fa-SS ← SS	1.369	0.193	7.097	***	0.834
MVPA-EE ← SS	2.115	0.408	5.183	***	0.328
MVPA-time ← MVPA-EE	11.656	0.227	51.444	***	0.938
MVPA-EE ← ST	−0.006	0.003	−2.273	**	−0.120

Regression Weights (R.W.); Standardised Regression Weights (S.R.W.); Standard Error (S.E.); Critical Ratio (C.R.); Moderate–Vigorous Physical Activity Time (MVPA Time); Social Support (SS); Screen Time (ST); Friend Support (Fr-SS); Family Support (Fa-SS); and Light Physical Activity Energy Expenditure (LPA-EE); *** *p* ≤ 0.001; ** *p* ≤ 0.05.

## Data Availability

Not applicable.
